# Clock gene *Per3* deficiency disrupts circadian alterations of gut microbiota in mice: *Per3* deficiency disrupts circadian alterations

**DOI:** 10.3724/abbs.2023257

**Published:** 2023-11-15

**Authors:** Xiaoxian Xie, Haoshen Xu, Ruonan Shu, Lei Sun, Mengya Zhang, Qinglian Hu, Kai Zhu, Zezhi Li, Fengchun Wu

**Affiliations:** 1 College of Biotechnology and Bioengineering Zhejiang University of Technology Hangzhou 310032 China; 2 Department of Psychiatry the Affiliated Brain Hospital of Guangzhou Medical University Guangzhou 510370 China; 3 Shanghai Mental Health Center Shanghai Jiao Tong University School of Medicine Shanghai 201109 China; 4 Department of Pharmacology University of Oxford Mansfield Road OX1 3QT Oxford UK; 5 College of Animal Science and Technology Northwest A&F University Yangling 712100 China

Circadian rhythms are present in all organisms. The endogenous circadian clock is an autonomous temporal system that generates a rhythm of approximately 24 h even in the absence of environmental signals. The “core” genes in the circadian clock system of mammals consist of *Clock* and *Bmal1*, which generate activators, along with *Cry1*, *Cry2*, *Per1* and *Per2*, which generate repressors. As one member of the Per family, *Per3* is not the “core” clock gene in the usual sense, but it is also essential for physiological regulation in mammals. *Per3* exhibits an obvious circadian pattern in peripheral tissues, which is important for the endogenous timing of specific tissues, but its variants have been linked to circadian preference, mental disorders, and cognitive responses to circadian dysregulation [1].

The intestinal microbiota is a complex microbial ecosystem consisting of many species of microbes that can produce metabolites to influence the health of the host and also can communicate with distal organs and impact the functions of organs. The microbiota is associated with maintaining metabolic homeostasis, processing and digestion of nutrients, and participating in the normal metabolism of lipids, which has a strong connection to the incidence of dysbolism, such as diabetes, obesity and cardiovascular disease [2]. It has been shown that the intestinal microbiome participates in the development of mental disorders via the gut-brain axis. Strikingly, the circadian clock was reported to impact microbial homeostasis, and daytime-dependent fluctuations were identified in fecal microbiota. These findings suggested that the gut microbiome shows diurnal rhythmicity in humans and laboratory animals. The metabolites produced by microbiota affect the expression of central and liver clock genes and further regulate lipid metabolism. The diurnal pattern of the microbiome is disrupted due to the mutation of the *Clock* gene, *Bmal1* knockout, and *Per1*/ *Per2* double mutation. However, the regulatory roles of noncore clock genes in microbiota rhythms are not well understood. Therefore, we investigated the impacts of *Per3* deficiency on alterations in the mouse intestinal microbiome in this study.

Male C57BL/6J mice were purchased from the China National Laboratory Animal Resource Center (Shanghai, China). Male and female *Per3* ^+/–^ mice were purchased from Nanjing Model Animal Center (Nanjing, China), and homozygous *Per3* ^–/–^ male mice were obtained by expanding the breeding. Mice were divided into six groups with equal numbers, and mice were killed every 4 h, where the onset of light was called ZT0 and lights-off was called ZT12. All animal experiments were carried out according to the protocols of Ethical Standards and Regulations authorized by Zhejiang University of Technology. First, the circadian rhythm expression of some phyla in the intestinal microbiome was tested by qPCR. Then, the cecal contents’ 16S rRNA genes V4 region in wild-type (WT) and *Per3* ^‒/‒^ mice of ZT4 and ZT16 group were sequenced with Illumina NovaSeq 6000 (Illumina, San Diego, USA) by GUHE Info Technology Co., Ltd. (Hangzhou, China). The collected raw data underwent analysis primarily using QIIME (v1.9.0) and R packages (v3.2.0). The process of assembling paired-end reads and selecting operational taxonomic units (OTUs) was performed by using Vsearch (v2.4.4).

**Figure 1:**
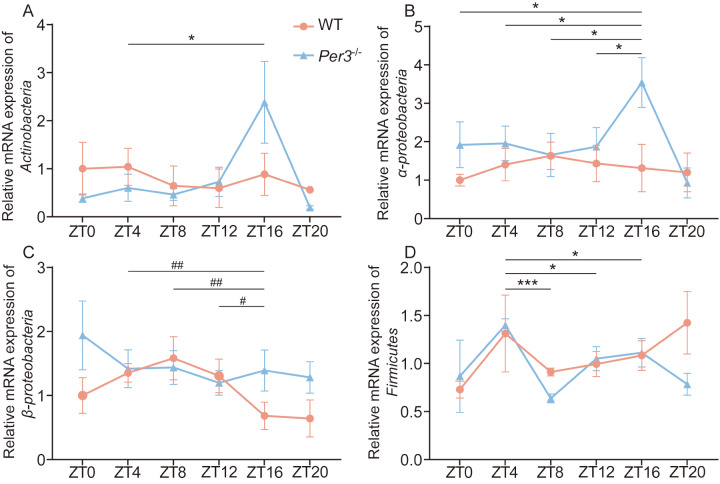
Figure 1 *Per3* deficiency altered the circadian rhythm of intestinal microbiome (A‒D) Relative mRNA expression of Actinobacteria, α-proteobacteria, β-proteobacteria and Firmicutes in WT and Per3 –/– group. #P<0.05, ##P<0.01, compared to the WT group. *P<0.05, ***P<0.001, compared to the Per3–/– group. n=5 in all groups.

**Figure 2:**
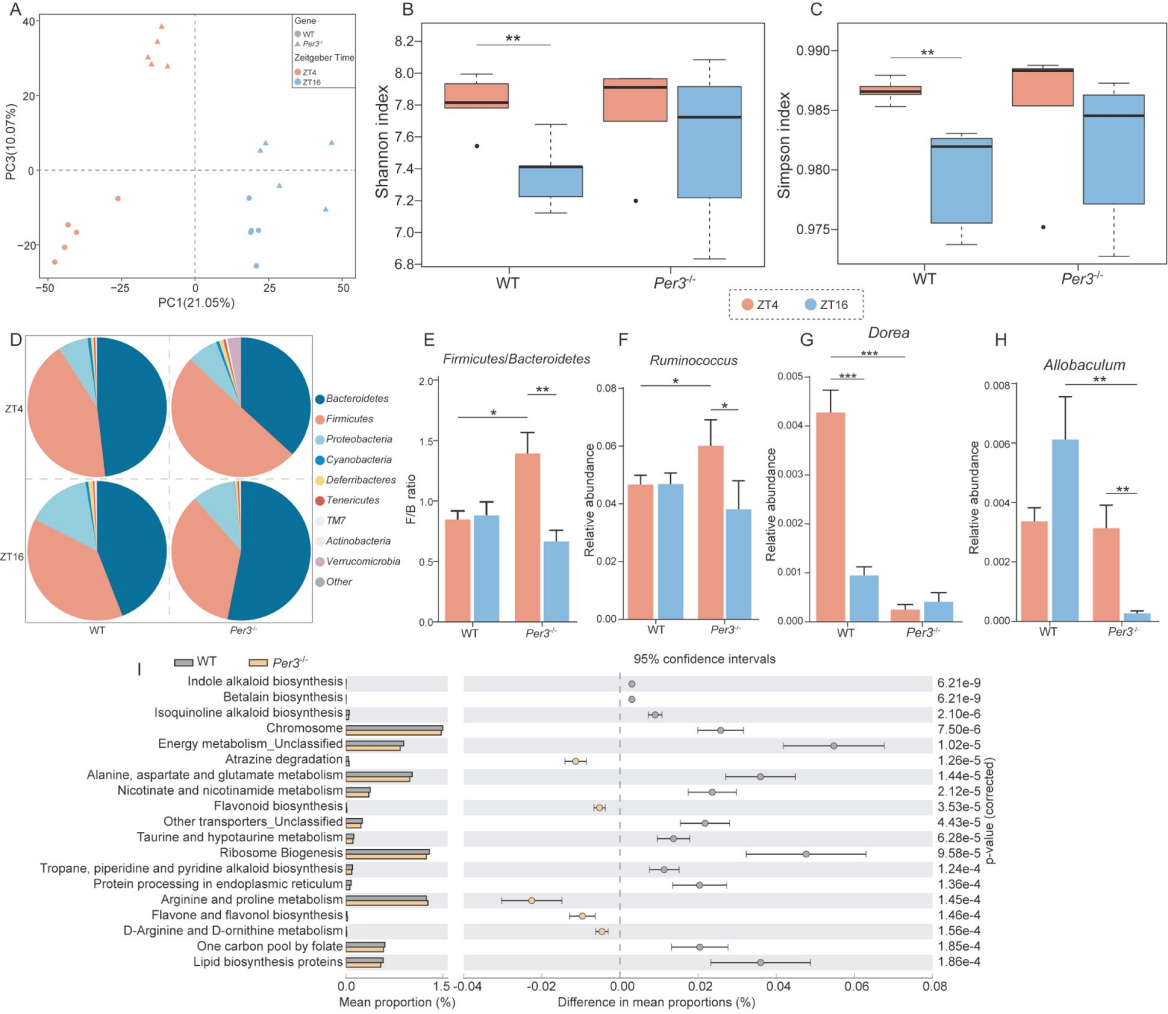
Figure 2 *Per3* deficiency caused the disorder of intestinal microbiome (A) PCA analysis of cecal contents in WT and Per3‒/‒ group. (B,C) Shannon index, Simpson index in WT and Per3‒/‒ group. (D) Relative abundances of the intestinal microbiome at the phylum level. (E) Ratio of Firmicutes to Bacteroidetes. (F–H) Relative abundances of Ruminococcus, Dorea, Allobaculum at the mRNA level. (I) KEGG enrichment analysis of intestinal microbiome. *P<0.05, **P<0.01, ***P<0.001. n=5 in all four groups.

The mRNA expression of *Actinobacteria*, *α-proteobacteria* , *β-proteobacteria* and *Firmicutes* showed that *Per3* deficiency altered the circadian rhythm of gut microbes ( Figure 1), in which the changes at ZT4 and ZT16 between WT and *Per3* ^–/–^ mice were significant. Furthermore, we analyzed changes in the cecal contents caused by *Per3* deficiency at these two time points. After distinct clustering, the composition of gut microbes showed a great difference between WT and *Per3* ^–/–^ mice, as well as between ZT4 and ZT16, using principal component analysis (PCA). Our data showed that the difference between WT and *Per3* ^–/–^ mice at ZT4 was larger than that at ZT16 ( Figure 2A). Both the Shannon index and Simpson index were reduced at ZT16 in WT mice compared to those at ZT4 ( Figure 2B,C); however, no change in the Shannon index and Simpson index was observed in *Per3* ^‒/‒^ mice across the same time points ( Figure 2B,C), showing a possible disruption of circadian rhythm caused by *Per3* deficiency. Then, specific changes in the composition at the phylum and genus levels were assayed. Increased relative abundances of the phyla *Firmicutes* , *Deferribacteres*, *TM7* and *Verrucomicrobia* were observed at ZT4 in *Per3* ^‒/‒^ mice compared to those in WT mice, and a reduced trend was observed for the abundances of *Bacteroidetes* and *Actinobacteria*. At ZT16, *Bacteroidetes* were increased, and *Proteobacteria*, *Verrucomicrobia*, *Cyanobacteria* and *Deferribacteres* exhibited a downward trend relative to controls ( Figure 2D). The *Firmicutes*/ *Bacteroidetes* ratio (F/B ratio) is generally recognized as a significant factor in maintaining microbiota homeostasis, and its variation is closely related to obesity, inflammatory bowel disease (IBD), hypertension and other diseases [3]. In the present study, we demonstrated that the F/B ratio was significantly elevated at ZT4 compared to that at ZT16 in *Per3* ^‒/‒^ mice, but no difference was identified in WT mice ( Figure 2E). At the genus level, the relative abundances of *Ruminococcus*, *Dorea*, and *Allobaculum* were significantly altered ( Figure 2F–H), which is partly consistent with observations in socially isolated mice [4]. The relative abundance of *Ruminococcus* showed a similar change trend to that of the F/B ratio, which is positively associated with the inflammatory response in IBD [5]. Additionally, *Ruminococcus* is associated with tryptamine metabolism, and its metabolites may influence the risk of developing neurological disorders. Lukić *et al* . [6] demonstrated that antidepressants reduced the abundance of *Ruminococcus*. The abundance of *Dorea* was significantly reduced in *Per3* ^‒/‒^ mice at ZT4 compared to that in WT mice, which tended to show a negative correlation with the severity of depressive symptoms. Together, our findings confirmed to some extent the roles of *Per3* in seasonal mood disorders in a previous report [7]. Moreover, the abundance of *Allobaculum*, which is known as an immunostimulatory species in gnotobiotic mice, was reduced in *Per3* ^‒/‒^ mice at ZT16 relative to that at ZT4 and in WT mice. A similar trend was observed in depressive mice, which exhibited a direct relationship with acetic acid and 5-HT [8]. In combination with the observations on *Dorea* and *Allobaculum*, our findings demonstrate that the altered composition of the gut microbiota caused by *Per3* deficiency may be linked to the risk of mood disorders. These observations implied that *Per3* deficiency may be associated with inflammation and mood disorders in rodents, which was supported by a previous report [7]. Additionally, KEGG analysis showed that *Per3* deficiency distinctively leads to alterations in nicotinate and nicotinamide metabolism, lipid biosynthesis proteins, indole alkaloid biosynthesis, energy metabolism and other pathways in mice ( Figure 2I). It is noteworthy that *Per3* deficiency may affect inflammation, lipid synthesis and mitochondrial homeostasis through the dysregulation of the intestinal microbiome and its circadian rhythm.

Internal circadian rhythms play an essential role in the function of different bodily organs to align environmental signals to achieve maximum efficiency. Recent evidence suggested that the gut microbiome has rhythmicity and exhibits diurnal variations. The biological rhythms of the gut microbiome depend on specific environmental cues within the gut, which fluctuate following dietary intake, host circadian rhythms, behaviors, and other various factors, such as melatonin and body temperature. What is becoming clear is that not all gut microbiota show diurnal oscillations in their abundance, in which small percentages are called microbial oscillators. These findings support our observations of gut microbiota changes in *Per3* ^‒/‒^ mice. Additionally, mutations in circadian clock-related genes have been identified as leading causes of gut microbiota rhythmicity disruption. However, the effect of *Per3* on the intestinal microbiome remains unclear.

Our findings demonstrate that *Per3* deficiency is related to the disorder of the intestinal microbiota composition and alterations in the rhythm system of the microbiome, particularly at ZT4. Similar phenomena were observed in the core clock gene deficiency mice, including *Per1*, *Per2* and *Bmal1*, suggesting that the compositions of gut microbes can be altered under conditions of clock gene deficiency in mice. There is extensive evidence suggesting that disruptions in the rhythm of the intestinal microbiome have a strong correlation with metabolic dysfunction, for example, the altered levels of secondary bile acids caused by disrupted rhythm of the microbiome in *Bmal1*-deficient mice, which was similar to those discovered in patients with type 2 diabetes and obesity [9]. These disruptions were also reported to be correlated with the development of neurological conditions. Sun *et al*. [10] revealed that cognitive impairment in mice can be alleviated by restoring the damaged intestinal microbiome with Dendrobium officinale polysaccharide. Mutations in the *Clock* gene lead to elevated anxiety-like behaviors in mice, which might be associated with disruptions in the circadian rhythm of the microbiome.

In conclusion, our results provide insights into the functional consequences of *Per3* on the disruption of the gut microbiome composition and may aid in improving our understanding of how the human gut microbiota is regulated by the circadian clock.
